# Isolation and Self-Association Studies of Beta-Lactoglobulin

**DOI:** 10.3390/ijms21249711

**Published:** 2020-12-19

**Authors:** Adrian Gołębiowski, Paweł Pomastowski, Agnieszka Rodzik, Anna Król-Górniak, Tomasz Kowalkowski, Marcin Górecki, Bogusław Buszewski

**Affiliations:** 1 Centre for Modern Interdisciplinary Technologies, Nicolaus Copernicus University in Torun, 4 Wileńska St., 87-100 Torun, Poland; adrian.golebiowski@doktorant.umk.pl (A.G.); p.pomastowski@umk.pl (P.P.); agnieszka.rodzik1@gmail.com (A.R.); annkrol18@gmail.com (A.K.-G.); tomasz.kowalkowski@chem.umk.pl (T.K.); 2 Department of Environmental Chemistry and Bioanalytics, Faculty of Chemistry, Nicolaus Copernicus University in Torun, 7 Gagarina St., 87-100 Torun, Poland; 3 Institute of Organic Chemistry, Polish Academy of Sciences, 44/52 Kasprzaka St., 01-224 Warsaw, Poland; gorecki_marcin@interia.pl

**Keywords:** β-lactoglobulin (β-LG), asymmetric flow field flow fractionation (AF4), oligomeric forms, protein stability

## Abstract

The aim of this study was to investigate isolated β-lactoglobulin (β-LG) from the whey protein isolate (WPI) solution using the column chromatography with SP Sephadex. The physicochemical characterization (self-association, the pH stability in various salt solutions, the identification of oligomeric forms) of the protein obtained have been carried out. The electrophoretically pure β-LG fraction was obtained at pH 4.8. The fraction was characterized by the matrix-assisted laser desorption ionization-time of flight mass spectrometry (MALDI-TOF/TOF MS) technique. The use of the HCCA matrix indicated the presence of oligomeric β-LG forms, while the SA and DHB matrices enabled the differentiation of A and B isoforms in the sample. The impact of sodium chloride, potassium chloride, ammonium sulfate, and sodium citrate in dispersion medium on β-LG electrophoretic stability in solution was also studied. Type of the dispersion medium led to the changes in the isoelectric point of protein. Sodium citrate stabilizes protein in comparison to ammonium sulfate. Additionally, the potential of capillary electrophoresis (CE) with UV detection using bare fused capillary to monitor β-LG oligomerization was discussed. Obtained CE data were further compared by the asymmetric flow field flow fractionation coupled with the multi-angle light scattering detector (AF4-MALS). It was shown that the β-LG is a monomer at pH 3.0, dimer at pH 7.0. At pH 5.0 (near the isoelectric point), oligomers with structures from dimeric to octameric are formed. However, the appearance of the oligomers equilibrium is dependent on the concentration of protein. The higher quantity of protein leads to the formation of the octamer. The far UV circular dichroism (CD) spectra carried out at pH 3.0, 5.0, and 7.0 confirmed that β-sheet conformation is dominant at pH 3.0, 5.0, while at pH 7.0, this conformation is approximately in the same quantity as α-helix and random structures.

## 1. Introduction

β-lactoglobulin (β-LG) is a small globular protein from the lipocalin family. It is constituted from 162 amino acid residues. The mass of the monomer is about 18.3 kDa [1]. It is the most abundant bovine whey protein, accounting for more than 50% of the total whey protein [2].

β-LG is an important source of the essential and branched-chain amino acids (leucine, isoleucine, and valine). This protein possesses antioxidant properties, because it contains two disulfide bonds (Cys-66 to Cys-160, Cys-106 to Cys-119) and one free thiol group (Cys-121) [3]. β-LG is a ligand transport agent. In particular, the affinity to hydrophobic compounds is significant due to the β-barrel structure [4,5,6].

As a result of whey processing, whey protein hydrolysate (WPH), whey protein concentrate (WPC), and the whey protein isolate (WPI) can be obtained. The WPH can be obtained by heating with acid [7] or enzymatic treatment [8]. β-LG is particularly sensitive to trypsin digestion [9]. The degradation and agglomeration temperature can be achieved at the range of 65–68 °C, depending on the pH and ionic strength [10]. The enzymatic hydrolysis of whey proteins liberates fragments that may contribute to the improvement of functions of the immune, cardiovascular, nervous, and gastrointestinal systems [11]. In contrast, WPC can be obtained as a result of modern membrane-based separation technologies such as ultra-filtration for protein concentration, diafiltration (DF) to remove lactose, minerals and low molecular weight compounds. Depending on their concentration, there are WPCs containing 35%, 50%, 65%, and 80% (*w*/*w*) of proteins. WPI with 90% (*w*/*w*) of protein is considered as a high quality and pure protein concentrate [12].

WPI is a good source of high quality native β-LG. It is important in food and biotechnology [13]. β-LG isolation can be performed by many routes [14,15], e.g., the precipitation by denaturing agents (salts, acids, acetone, temperature). However, these approaches can also lead to undesirable precipitation of other proteins. Column chromatography is a technique that allows for isolation of high purity protein, but the selection of experimental conditions is crucial. β-LG occurs in several isoforms, where the A and B isoforms are dominant [16].

In solution β-LG exists in different oligomeric states, which depend on the pH, the ionic strength, temperature, and protein concentration [17]. The main factor contributing to the oligomerization of β-LG, taking into account the effect of variable pH and ionic strength on dimerization, is the hydrophobic effect. According to theoretical calculations, the hydrophobic effect is the result of a decrease in the area accessible to water when dimers are formed [18]. At room temperature, β-LG occurs mainly as a dimer in unprocessed milk, but below pH = 3.5, β-LG becomes a monomer. Isoform β-LG A forms octamers close to pH = 4.6 at temperature below 20 °C, while β-LG B is less resistant [19].

The circular dichroism spectra recorded in far UV region (far UV-CD) can solve the secondary structure of proteins. Based on the CD spectra, α-helical proteins possess negative bands at 222 nm and 208 nm and a positive one at 193 nm. Proteins with β-sheet conformation have negative bands at 218 nm and positive bands at 195 nm. In turn, the random conformed proteins have very low ellipticity above 210 nm and one band near 195 nm [20]. The main conformation of β-LG is β-sheet at pH close to physiological [4,21,22,23,24,25,26]. Oligomeric state is dependent in particular on pH; at pH 2.0 it is a stable monomer, while at neutral pH it shows a dimeric state. In strong acidic conditions β-LG chains are highly positively charged, and they repel each other. In neutral pH conditions, the dimeric state is stabilized by hydrogen bonds between the surface AB loop and the anti-parallel β-sheet [27].

Different values of 5.1 [28], 4.8 [29,30], and 5.2 [31] were reported for the isoelectric point of β-LG. The differences can be caused by solvent components, the ionic strength, employed analytical method and fitting of experimental data. The influence of various trivalent cations on the protein surface charge was discussed in literature [32]. The authors showed that multivalent cations could change the protein surface charge more effectively than monovalent cations.

The zeta potential analysis is the most popular technique for determination of the surface charges of biocolloids and investigation their stability in the fluids. It is also used to determine the isoelectric point of colloid particles. The advantages of this technique are connected to the possibility to perform the zeta potential and isoelectric point measurements in a wide range of pH, ionic strength and study of the impact of buffer composition on these parameters. In the case of protein research, the impact of buffer components on protein physicochemical stability and isoelectric point values seems to be the most important. Other techniques like isoelectric focusing (IEF) and capillary isoelectric focusing (CIEF) do not allow for determination of the stability at different pH values, ionic strength, and composition of solvent. However, these techniques are advantageous for high resolution and short time of analysis [33].

The association of β-LG protein has been studied using analytical ultracentrifugation or small angle X-ray scattering [18,34]. However, there are still many questions regarding this process and the factors affecting protein oligomerization. Thus, a new interdisciplinary approach for deeper characterization of β-LG forms is needed. Capillary electrophoresis (CE) is one of such techniques and provides a rapid and efficient protein analysis with a low sample and buffer consumption [35,36]. The technique is typically applied for protein separation [37,38,39], but recently the use of CE as a screening method for the protein oligomers determinations is attracting attention [40,41]. Therefore, the potential use of CE for rapidly monitoring of β-LG forms at different pH conditions was studied in this paper.

Light scattering techniques can estimate the molar mass of the particles and thus distinguish protein forms in solution. Moreover, the multi-angle light scattering (MALS) detector coupled to the separation technique gives information about the molar mass distribution, the radius of the gyration of polydispersed protein forms. The asymmetric flow field-flow fractionation (AF4) gives opportunity for a “soft” separation with minimal changes of the sample composition during analysis [42]. This approach was developed in the mid-1960s by Giddings [43,44]. Today, the application of the technology is more appropriate for separation of large, highly branched colloids in comparison with other techniques [45]. The oligomeric states and molar mass of α-lactalbumin complexes were successfully investigated by the AF4-MALS technique by S. Dhayal et al. [46]. The formalism used to calculate the particle size and the average weight of molar mass has a crucial impact on the results [47] Proteins, therein the β-LG, were analyzed by the AF4 technique [48]. The authors also discussed a selection of the channel membrane and analyzed an overloading effect.

In turn, a powerful tool for protein identification is the matrix-assisted laser desorption ionization-time of flight mass spectrometry (MALDI-TOF/TOF MS) technique, characterized by high sensitivity, low detection limit (LOD), and simplicity of use. The MALDI-TOF/TOF MS method allows for a simple determination of protein masses, as well as analysis of the peptide fragmentation and localization of the posttranslational modifications of proteins [49].

The aim of this study was to investigate the isolated β-LG from the WPI solution, applying column chromatography. Characterization of the β-LG was carried out by multi-instrumental approaches. The data on the factors affecting the protein characterization has not been thoroughly discussed in the literature. Thus, obtained data presented in the current study shed light on β-LG self-association, which seems to be crucial not only for analytical chemistry, but also for the dairy industry. It shows potential to affect the processing and manufacturing of milk products. In this context, the different physicochemical parameters such as the impact of MALDI-TOF/TOF-MS matrix, solvent composition and pH were tested. The dispersion stability and oligomeric forms of the isolated β-lactoglobulin were characterized by the use of zeta potential measurements, capillary electrophoresis, and asymmetric flow field-flow fractionation (AF4) with the multi-angle light scattering (MALS) detector technique. This innovative complementary approach allowed for detection of oligomeric state of the β-LG at pH 3.0, 5.0 and 7.0. Furthermore, we investigated conformation of protein in these conditions by applying circular dichroism (CD) spectroscopy.

## 2. Results

### 2.1. Isolation of β-LG from WPI Solution by Chromatographic Column

**Figure 1:**
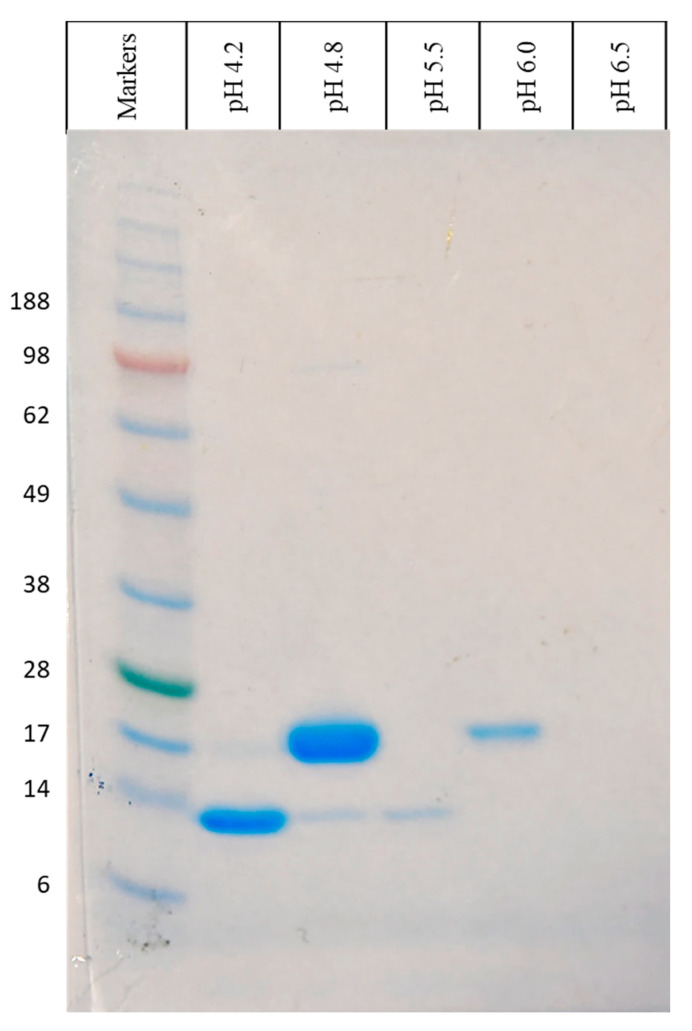
SDS-PAGE electropherogram at reduced condition of fractions from WPI separation.

β-lactoglobulin was isolated in a 0.2 M citrate buffer at the linear pH gradient (from pH 3.0 to 6.5) by column chromatography. The isolation method was optimized by changing the pH of eluent. The first set up scheme was created using a pH gradient from 3.0 to 6.5 with the increment of 0.5 pH units. The pH 5.0 fraction was enriched mostly with β-LG. However, α-lactalbumin was also collected from the same solution (data not shown). Thus, the pH values of buffer at which fractions were collected were set to 4.2, 4.8, 5.5, and 6.0; consequently, one single band of proteins was observed in the range of 14 and 17 KDa (Figure 1) and fraction at pH 4.8 was mostly enriched by β-LG. The 14 kDa protein was found in this solution, which can be treated as impurity. However, the intensity of the impurity was low, and it could be seen with utilization of proteins solution of particular fraction with high concentration. The 301.3 mg of dry β-LG was from 0.1 L of WPI concentration of 10 g/L.

### 2.2. Characterization of β-LG by MALDI-TOF/TOF-MS Analysis

In order to characterize β-LG by MALDI-TOF/TOF MS spectrometric method, isolated fractions of WPI at pH values: 4.5, 5.0, 5.5, 6.0, and 6.5 were subjected to electrophoresis in the polyacrylamide gel. Gel electrophoresis was used to determine the purity of protein in the sample. The obtained bands were obtained due to the separation of protein on the basis of the charge-to-weight ratio and indicated the presence of β-LG at about 18 kDa.

MALDI-TOF/TOF MS analysis of intact proteins carried out with two matrices: HCCA and SA. Their application allowed for observation of the presence of β-LG only in the fraction at pH 4.5 for both matrices (Figure S1) In case of both matrices used, the presence of α-lactalbumin (α-LA), which is the second major protein in whey after β-LG, was also observed for the fraction at pH 4.5. For the HCCA matrix, α-LA was also present in the fraction at pH 5.0 and with SA matrix additionally in the pH 5.5 fraction. No proteins were found in the remaining pH 6.0 and pH 6.5 fractions.

Comparing the utilization of both matrices, it was found that it is possible to observe isoforms of the protein with sinapic acid. Masses obtained for the protein with application of HCCA and SA as matrices using the MALDI/TOF MS by the intact approach are shown in Table 1.

More detailed investigations of the β-LG fraction components were carried out after their tryptic digestion, after which the fingerprints obtained in the positive reflection mode were analyzed. The mass spectra of tryptic peptides obtained for the fraction collected for β-LG are presented in Figure S2 and Table 2.

### 2.3. Characterization of β-LG Zeta Potential

**Figure 2:**
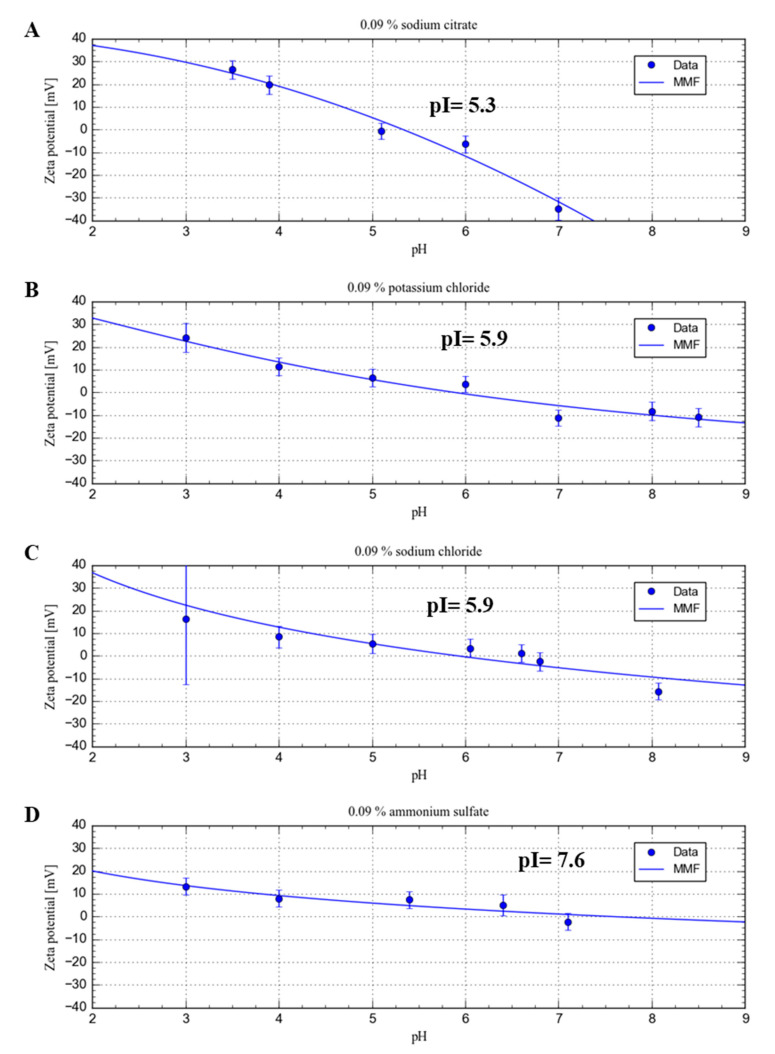
The pH dependence of the zeta potential at 0.09% (*w*/*v*) sodium citrate (**A**), potassium chloride (**B**), sodium chloride (**C**), and ammonium sulfate (**D**). The blue line represents fitted line to the experimental data (sigmoidal fitting, MMF function).

The zeta potential was investigated in the pH stability range of solvent component and protein. Figure 2 depicts pH the dependence of the β-LG zeta potential dispersed in several media with concentration 0.09% (*w*/*v*): sodium citrate, sodium chloride, potassium chloride, and ammonium sulfate. The zeta potential dependences on four solvent compositions were typical for proteins.

At a pH value lower than the isoelectric point (pI), the zeta potential was positive. In contrast, at a pH value higher than pI, the zeta potential was negative. The highest positive values of the zeta potential were observed in pH 3.0, and the sodium citrate buffer accounted for 26.4 ± 4.1 mV. In that solution, the standard deviations of the zeta potential were also found to be the lowest. The lowest value of the zeta potential at pH 3.0 was observed in the sodium chloride solution, but it was associated with the highest deviation. The isoelectric point of protein varied depending on the individual medium composition. In case of the sodium citrate buffer, it was estimated as 5.3. In turn, the higher value was observed for the other mediums. For example, in the ammonium sulfate solvent the pI reached 7.6. In the sodium citrate buffer, the protein had the lowest zeta potential value in alkaline condition −34.8 ± 4.8 mV. In the other medium conditions, the zeta potential was higher, reaching the zero value with a much higher deviation. The highest values of zeta potential deviation were recorded for the ammonium sulfate solvent in the whole pH range.

### 2.4. Characterization of β-LG Oligomerization by CE

**Figure 3:**
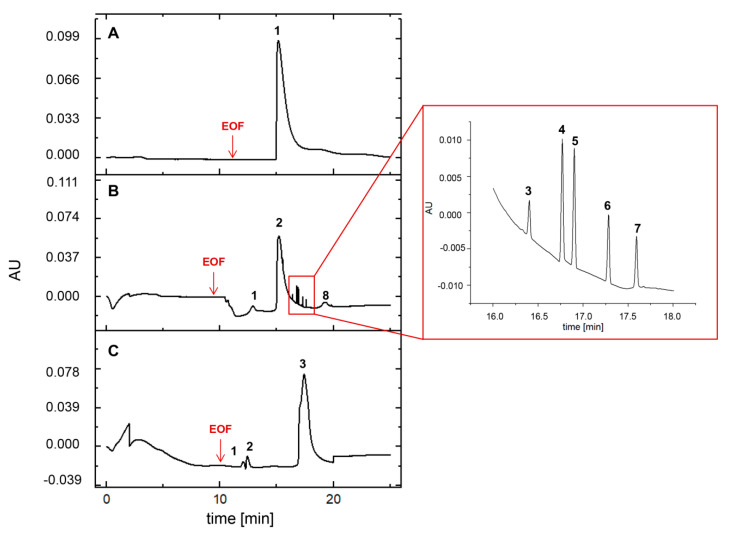
Electropherogram of β-LG at pH 3.0 (**A**), 5.0 (**B**), and 7.0 (**C**). The EOF acronym means the electro-osmotic flow. The compounds (oligomeric forms of protein and impurities) leaving the capillary were signed with subsequent numbers in each electropherogram.

The goal of the electrophoretic study was to monitor the β-LG oligomerization process. The Figure 3A–C shows the electropherograms of β-LG at pH 3.0, 5.0, and 7.0, respectively. The sample at pH 3.0 can be characterized by one CE signal zone (1) at the 15.16 min electromigration time (Figure 3A). In case of protein suspension in the buffer at pH 5.0 (1–8), the number of CE signals occurred at 8 (Table 3, Figure 3B). In contrast application of buffer at pH 7.0 resulted in the observation of three signals (1–3) (Table 3, Figure 3C).

The electrophoretic mobility (µe, cm^2^/Vs) value of β- lactoglobulin at pH 5.0 and 7.0 decreased within the CE analysis time (Table 3). Different buffer conditions affect the changes in electrophoretic mobility of β-LG, and consequently, its stability. According to the Smoluchowski equation [50], the zeta potential (ZP) depends proportionally on electrophoretic mobility of particular colloidal particles and inversely proportional to the dielectric constant of the medium. Since the ZP value is a crucial parameter for determination of long term stability of biocolloids. It has been assumed that ZP values between +30 mV and −30 mV typically demonstrate low degree of stability. In addition, deviation from these values provide higher stability of biocolloids [51,52]. Based on the obtained results, it can be concluded that β-lactoglobulin at pH 5.0 has a high tendency to oligomerization as well as creation of aggregates. In comparison with pH 3.0 and 7.0, electrophoretic separation of β-LG around its isoelectric point (pI) provides the highest number of signals, and consequently, lower electrophoretic mobility rate and dispersion stability were observed (Figure 3B, Table 3).

### 2.5. Characterization of β-LG Self-Association Using AF4-UV-MALS

**Figure 4:**
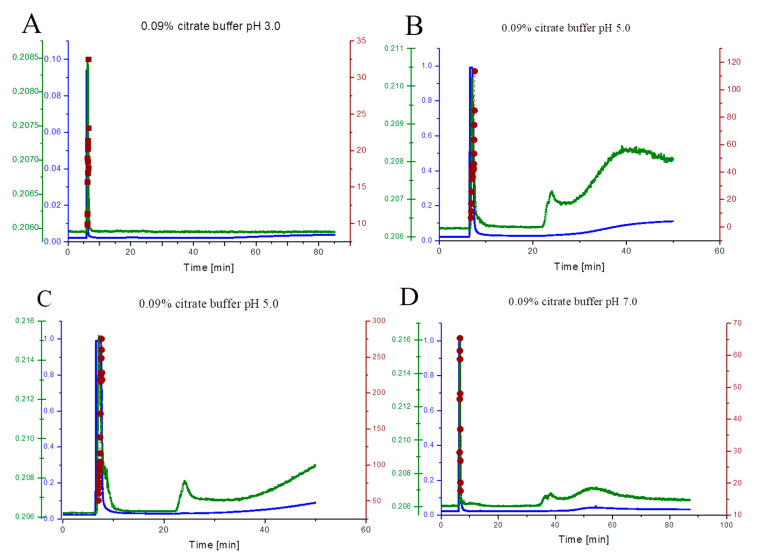
AF4-UV–MALS fractograms of β-LG in 0.09% (*w*/*v*) sodium citrate pH 3.0 (**A**), 5.0 (**B**,**C**), and 7.0 (**D**). The blue scale comes from UV detector (signal in V), green scale from MALS detector (92 angle, signal in V), and brown scale indicates for weighted-average molar mass (kDa).

The β-LG was characterized by multi-angle light scattering technique in view to investigate the molar masses (molecular form) in 0.09% sodium citrate buffer at three pHs: 3.0, 5.0, and 7.0. To achieve this goal, the AF4 was coupled to the MALS detector. Figure 4 presents the acquired fractograms. The normal mechanism of separation was obtained for all tested conditions. The mass of the first fraction was dominant for all the samples in the whole pH range.

At pH 3.0, two fractions were recorded. The first fraction, obtained between 5 and 6 min of the analysis, had a mean weight average molar mass of 22.1 ± 0.5 kDa (Figure 4A, Table 4). The second fraction was registered as a weak signal, appearing from about 55 min of the analysis.

Similarly, at pH 5.0, two fractograms were shown (Figure 4B,C). Both of them present three fractions of protein particles. The Figure 4B shows the particles with a mean weight average molar mass of 50.2 ± 12.5 kDa, while the Figure 4C indicate the presence of particles with a three times higher mass, reaching the mean at 149.0 ± 13.7 kDa. The first fraction was eluted just after the focusing step (from 6.5 min to 8 min of the analysis); the second fraction was eluted after 25 min of the analysis.

At pH 7.0, three fractions were recorded. The first fraction was eluted in the relaxation step (from 6 to 8 min of the analysis). Particles had a mean weight average molar mass of 38.7 ± 8.2 kDa. Another fraction is acquired at the elution step (from 45 to 65 min of the analysis) (Figure 4D).

At pH 5.0, high polydispersity in the first fraction was observed. The second and third fractions represent agglomerates of protein. The molar mass of these species was in the range of 10^5^–10^7^ kDa.

Figure S6 presents the example SDS-PAGE result from the analysis at pH 3.0. One single band between 14 and 17 kDa was observed. The band from the first fraction was the most intensive. The sample filtrated by the Amicon with the membrane cut off value of 10 kDa also showed bands at the aforementioned range. The results from gel electrophoresis at pH values of 5.0 and 7.0 are presented in Figure S7. At pH value of 5.0, one single band from the first fraction is observed with the range from 14 to 17 kDa, while no bands for the second and third fraction were observed. At pH 7.0, one single band is present at the aforementioned range for the first and third fractions. In case of the second fraction no bands were observed.

### 2.6. Characterization of β-LG Fraction from AF4-UV-MALS Using MALDI-TOF/TOF MS

The fractions from the AF4-MALS analyses were collected into Eppendorf tubes, concentrated and subjected to the gel electrophoresis as well as the MALDI-TOF/MS analysis in the intact protein mode with utilization of HCCA and DHB matrices (Figures S3 and S4).

Using the HCCA matrix for the obtained three fractions for pH 3.0, the appearance of dimers, trimers, tetramers, and pentamers (in the case of the first fraction) was observed. When the pH increased to 5.0, the same was observed for the first fraction, while no signals were recorded for the second and third fractions. A slightly different situation was observed at pH 7.0; namely, the presence of dimers, trimers, tetramers, and pentamers was observed for fraction one.

For the second fraction, similarly to the applied pH 5.0, no signal from the β-LG was observed, whereas for the third fraction the presence of dimer was detected. On the other hand, the use of the DHB matrix allowed for observation of similarity in the appearance of oligomers for individual fractions at pH 5.0 and 7.0. For pH 3.0 only, the presence of peak corresponding to the β-LG monomer was observed. Additionally, the DHB matrix compared to the HCCA matrix indicated the presence of two genetic forms (isoforms) for each pH. The β-LG masses obtained for the three fractions from the AF4-UV-MALS using the MALDI-TOF/TOF MS are shown in Table S1.

In addition, for three fractions of pH 3.0 obtained by the AF4-UV-MALS, MALDI-/TOF MS were analyzed on diluted (1:10) β-LG samples. Significant differences were observed in comparison with more concentrated samples. For both HCCA and DHB matrices, the β-LG was detected only for the first fraction. When the HCCA matrix was used, the presence of β-LG isomers was found, whereas when the DHB matrix was used, two separate β-LG signals distinguishing A and B β-LG formulas were found (Figure S5).

### 2.7. Characterization of β-LG Secondary Structure by Far UV-CD

**Figure 5:**
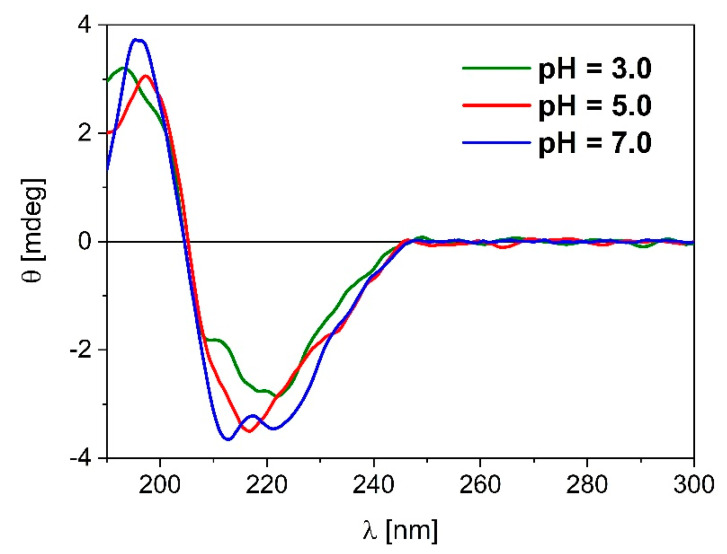
ECD spectra of β-LG carried out at pH 3.0, 5.0, and 7.0 in 0.09% citrate buffer.

The circular dichroism (CD) spectra obtained in far UV range are presented in Figure 5. Due to the absorbance of citrate buffer, the CD signal was obtained up to 190 nm; thus, the range from 190 to 250 nm were taken into consideration for analysis of the secondary structure. The differences are present according to pH values at which β-LG is dissolved. In the secondary structure range at pH value of 3.0, β-LG has a positive bands at 193 nm and two negative bands at 208 and 222 nm. In value of pH of 5.0, the protein structure exhibits a positive band at 197 nm and negative band at 217 nm. In contrast, at alkaline condition (pH of 7.0) a positive band is observed at 196 nm and two negative bands are obtained at 212 and 221 nm.

## 3. Discussion

Whey proteins comprise the fraction of ruminant’s milk and particularly bovine milk that is soluble at pH 4.6 [53]. They constitute about 20% of the total mass. The β-LG is the most abundant protein in bovine whey. The average concentration of the β-LG in whey is 3–4 g/L [14], but the isolated amount depends on both the procedure and the initial quantity in the milk. The yield of the isolation β-LG was 56%, taking into account the proteins amount in the WPI equal to 90% (according to the manufacturer specification) and the quantity of β-LG in the mass of protein equal to 60%.

Gel electrophoresis analysis was performed for the initial identification of isolated proteins and assessment of purity of the obtained fractions. Single fractions are the electrophoretically pure grade protein solutions (>95%). The comparison of the sample and protein marker bands can be used as a screening method for protein identification. In this way, one can state that the fraction at pH 4.2 is α-lactalbumin. This protein has a monomer form with the molecular mass of about 14 kDa, and it is the second most abundant protein in whey. The fraction at pH 4.8 is a solution of β-LG. The co-elution of α-lactalbumin with this protein was observed, but the concentration effect exists. The isoelectric point of these two proteins is closely localized at the pH scale (pI of α-lactalbumin is about 4.2–4.6) [54]. The optimal selectivity of separation was achieved by a slight pH increase (from 4.0 to 4.2) to elute α-lactoalbumin and a slight pH decrease (from 5.0 to 4.8) to obtain the elution of β-LG. Fractions that are collected at higher pH contain a smaller amount of proteins. To confirm this screening, identification by the MALDI-TOF/TOF MS analysis was performed.

The MALDI-TOF/TOF MS analysis is currently a widely used technique in proteomic research [55].

A key aspect of MALDI-TOF/MS analysis is the matrix. The use of different matrices allows for different protein coverage as well as slight differences in the obtained protein molecular weights [56]. Two matrices were used in this study: HCCA and SA to characterize β-LG obtained by column chromatography as a control in relation to the MALDI-TOF/MS analysis for β-LG after the AF4-UV-MALS analysis. The chemical structure of the applied matrices consists of a benzene ring that absorbs UV light, hydroxyl groups for simplified mixing with hydrophilic biomolecules, and a carboxyl group that acts as a proton donor [57]. The structural details of the matrix molecule, e.g., the hydrophobicity and hydrophilicity of the compound, exert a great effect on the degree and extent of protein ionization in the matrix-assisted laser desorption [58].

The obtained MALDI-TOF MS spectrum for intact β-LG with the SA matrix showed two peaks with *m*/*z* 18 301.1 ± 0.0 and 18 382.8 ± 0.0 (Figure S1), which can correspond to β-LG B and β-LG A genetic variants [59]. The difference in masses of β-LG A and β-LG B can arise from the substitution of the amino acids such as Asp in position 64 instead of Gly and Val in position 118 instead of Ala [59]. Furthermore, β-LG A showed a higher intensity than β-LG B, indicating a higher content of β-LG A in the sample. Similar was observed using the SA matrix by Hemung et al. reporting higher intensity to β-LG A [60].

In addition, the detected octamers are assigned to be the β-LG A form [61]. Isomers A and B were also observed for α-LA at pH 4.5, 5.0, 5.5. Variant A compared to variant B differs in the presence of Gln at position 10 instead of Arg.

In the case of the β-LG characterization using the HCCA matrix, only one monomeric form was observed.

For the characterization of β-LG AF4-UV-MALS fractions using the MALDI-TOF/TOF MS, the HCCA and DHB matrixes were used. The use of the HCCA matrix allowed obtained of the mass spectrum indicating the presence of the monomer as the main peak and small peaks corresponding to oligomeric β-LG forms. However, the use of DHB matrix, apart from indicating oligomeric forms, indicated the presence of β-LG isoforms.

It is claimed that the absolute zeta potential of the investigated system greater than ±25 mV suggests that the colloidal particles would remain in a stabile dispersion state [62]. The electrostatic repulsion constitutes the next force for the steric mechanism, considering the stability of the dispersed particles in the medium. Unfortunately, at extremely low or high values of pH, the protein can degrade its quaternary structure, losing its biological activity [62]. For this reason, the zeta potential measurements were carried out in a stable range of protein and solvent components. The concentration expressed in mass units (mass/mass or mass/volume) in this study was used. This form of concentration units is frequently used in proteomic studies for proteins as well as dispersants. Therefore, we compare data with other works carried out for various proteins in our lab [55,62,63]. However, this expression of concentrations implies various molar concentrations for different salts component and consequently various ionic strength for dispersants. Therefore, direct comparison of ZP obtained data between them is not adequate.

The positive value of the zeta potential is the result of the protonation of carboxyl and amino groups from amino acid residues. The protonation is the strongest at the lowest pH investigated. However, only in citrate buffer, the zeta potential of β-LG is higher than the value for stabile dispersion. In another medium, the electrostatic stability was lower.

Deviation of zeta potential is also a significant mark of protein stability, because it is a derivate of polydysperity, impurity content, and inherently instability of sol [64] Very high deviation in sodium chloride at pH 3.0 was observed most preferably due to the degradation of protein at acidic conditions. At higher pH, the deprotonation took place and consequently lower zeta potential values were observed. According to the Hofmeister series and our results, it is clearly shown that the protein has different stability in the suspension. The strongest stabilizing agent is citrate buffer, followed by potassium chloride, ammonium sulfate, and sodium chloride. At higher pH values, the lower repulsion forces and the higher attracting forces between particles are present. The stability of protein dispersion depends on neutralization of protein charges due to specific adsorption properties to protein surface. Citrate anions have high affinity to protein cationic junction zones [65]. Another physicochemical parameter, which must be analyzed, is hydration potential of ions. Strong hydrated cations (in this case sodium in comparison to potassium and ammonium) create a highly stabile complex with carboxylic moieties of glutamic and aspartic residue. In contrast, weakly hydrated anions bind hydrated cations tightly then strongly to amide groups [66].

The pH at which the particle has equal amount of positive and negative charged residues is called the isoelectric point (pI) [67,68]. Consequently, the particles reach pI, when the Stern layer has the same electric potential as the diffusive layer of particle. The solvent components discriminate pI of β-LG. The DLVO theory assumes that the type of counterion and the valences have impact on the diffuse layer. Positive zeta potential values appear when the anions of salts are adsorbed onto the protein particle, and they constitute the Stern layer. Counterions have a variable screening effect on the protein.

The repulsive forces are lower when the higher valance ions are present in the solution. Chloride is a single negative charged species, but citrate anion is higher in size and depending on pH can have charge from minus one to three. The citrate anion is known as a complexing protein agent. Thus, the citrate ions can stabilize the protein by electrostatic and steric mechanisms. In this way, citrate anion stabilizes more evidently the β-LG especially close to its electric equivalence. According to the electro kinetic theory, the higher valance ions bind more strongly to the protein surface than the monovalent ones and neutralize the protein charge more effectively [69]. The thicker Stern layer has impact on the diffuse layer. Thus, the pI is achieved for higher valance ions at more alkaline pH [70]. Światek et al. [71] discussed relationships between the zeta potential and pH in various ionic strength and buffer compositions.

On the other hand, after exceeding the isoelectric point, the protein net charge is negative. In this case, the cations are counterions. In this work, sodium, potassium, and ammonium cations were assessed. For alkaline solution citrate anion is triple negative charged, and consequently sodium cation has a marginal effect, because the diffusive layer is thin, in comparison with the system when chloride is present as an anion. Sulfate anions are double negative charged species in the entire measured pH range [72]. The single charged ammonium ion also has the lower impact on the zeta potential of protein. Nonetheless, based on the obtained results, one can conclude that the cations in the solution have very low impact on the zeta potential of protein, because their influence is screened by multi charged anions. The zeta potential in the alkaline range is similar, with the exception of triple negative citrate buffer.

The self-association of the bovine β-LG was extensively studied by several physical techniques in the past [73,74].

In our study, capillary electrophoresis with the UV detection was able to separate a few different species of β-LG at pH 5.0. According to electrokinetic theory and based on the electrophoretic mobility, the order of migration is consistent with the β-LG mass. Then, it can be concluded that at pH 5.0 examined protein creates both diverse oligomeric forms (from dimers to octamers) (1–2 and 8 at Figure 3B) as well as the aggregates (3–7 at Figure 3B). On the other hand, the separation at pH 3.0 and pH 7.0 resulted in one and three signals, respectively (Figure 3A,C). Considering the phenomenon called the Tanford transition [18], the β-LG protein at pH close to physiological undergoes reversible conformational change and is mostly dimeric. Mercadante et al. [18] have demonstrated that an increase of the ionic strength strongly promotes the formation of the β-lactoglobulin dimer. Consequently, bovine β-LG is likely to be dimeric at the pH typically associated with milk [75]. However, CE data from our study also showed the formation of one homogenous β-LG fraction at the pH 3.0 (peak 1 at Figure 3A)—and it seems that the protein exhibits similar oligomerization behavior as at pH 7.0. However, the UV-CE detection is not sufficient to distinguish oligomeric forms under these conditions; hence, it was necessary to implement another approach with the AF4-UV-MALS technique.

Our results at acidic conditions show the monomeric state of β-LG, in contrast to the alkaline environment, where β-LG exists as a dimer [18]. Genetic variants β-LG A and β-LG B show structurally high resolution in a monomeric and dimeric state. For the β-LG A variant at low temperatures <10 °C, moderate ionic strength about 0.1 M, and near the isoelectric point about pH, 5.3 there is a low resolution of structural evidence for octameric form about 144 kDa. In contrast, for genetic variant β-LG B, no oligomerization of higher order is observed [18].

At pH 3.0 in the 0.09% sodium citrate dispersion, the monomer form was dominant. The mean weight average molar mass is quite consistent with the theoretical value. At pH 5.0, the results are unclear, because they indicate various forms of the protein in this condition. Additionally, the separation technique did not isolate these forms onto individual fractions. However, one can state that at pH 5.0, the protein creates from dimer to octamer forms. Two sorted series of the result are shown. The first series indicate the presence of a dimer, trimer, and tetramer form. The second series shows the octamer form. It means the β-LG has dynamic equilibrium between these species and the presence of these individual is not dependent on the pH, ionic strength, and temperature, but the difference in the protein concentration can be decisive. The higher content causes the octamer formation to depend on the concentration in the channel [42]. Thus, under this step, the particle concentration near the membrane rises to maximal values with much bigger content than in the stock solution. This local compacting can enhance the self-association of the β-LG. In turn, lower content entails to the formation of lower associate species. Beside the pH dependency, the ionic strength has a strong impact on the stabilization of the forms [76]. A higher content of salts stabilizes dimer [73], but when exceeding a certain value, it leads to the monomer formation. According to literature, our results confirm the occurrence of the dimeric form of the β-LG at pH 7.0 [73].

In all the analyses, the agglomerate fractions are detected. In reference to the zeta potential results, the repulsion–attraction forces are always present. Citrate ion stabilizes protein effectively in the acidic condition, so the only small amount of aggregate was detected. On the other hand, citrate also has significant reducing capability [77]. At pH close to isoelectric point, the attraction forces are dominant, and consequently, the aggregate is created rapidly [78]. The two fractions of agglomerate are presented. The formation of the agglomerate takes place on the diffuse barrier. At pH 7.0, two fractions of the agglomerate are also detected.

The electrokinetic theory does not explain that phenomena, because a strong negative value of the zeta potential was measured. The creation of the stabile complexes of protein with the citrate ion is the possible reason. Marioli and Kok analyzed the β-LG in 0.15 M PBS buffer at pH 7.2 using AF4 [48]. Fractogram also contained two fractions: dimers were reported in the first fraction. However, authors did not discuss the source of the less intensive second fraction.

The electropherograms (as a control) show that only the β-LG was detected after the separation process. The protein did not degrade under the analysis conditions.

We carried out the CD analysis to obtain information about the main conformation(s) of our system at pH values of 3.0, 5.0, and 7.0. This analysis was supported by the Secondary Structure Estimation (SSE) software from JASCO based on the Principle Component Regression (PCR) method. In acidic condition, the obtained signals indicated mainly β-sheet conformations (38%) with similar amount of random coil structures and some amounts of α-helical conformation (13%). In turn, in pH value of 5.0, β-sheet conformation is still dominant (35%), while the amount of α-helical structure increased about twice (25%). Finally according CD spectra, at pH of 7.0 α-helical, β-sheet and random conformations are approximately at the same amount (*ca.* 30%) in equilibrium of 0.09% citrate buffer. These results are in accordance with works cited in introduction section. The main conformation of β-LG is antiparallel β-pleated sheets with some content of α-helical conformation [26]. Although cited works were focused on neutral and alkaline pH environment (pH 6–9) where β-LG possesses dimeric structure, Wada et al. report β-sheet conformation and no noted differences between pH dependence (5 mM sodium phosphate buffer, pH 7.5 and 5 mM acetate buffer, pH 3.0) [22]

## 4. Materials and Methods

### 4.1. Protein Isolation from WPI Solution

WPI was purchased from the Dairy Cooperative Spomlek (Radzyń Podlaski, Poland). The isolation method for the fractionation of protein from the WPI’s solution was followed by the Etzel procedure [79] with minor modifications and optimization. Elution solutions have been optimized by small change of pH in comparison with the original patent. In our study, this small change leads to increasing the purity fractions.

Firstly, a 10 g/L solution of the WPI in 0.2 M citrate buffer pH 3.7 was prepared. The solution was adjusted to pH 3.0 against pH meter. Afterwards the soluble protein fraction was clarified from curds, lipids, and other insoluble parts using a centrifuge (14,400 rpm, 1 h, T = 4 °C). After that, the solution was mixed with SP Sephadex C-25 (GE Healthcare Bio-Sciences AB, Sweden), the resin of which was swelled and conditioned prior to separation. The mass ratio of WPI to dry mass of the ion exchanger was 0.33. The chromatographic column was packed with about 40 mL of slurry at room temperature. After washing with water, the elution procedure was performed with increasing pH (from 3.0 to 6.5) conditions. Additionally, the buffer solution at pH 3.0 consisted of 0.02 M EDTA, while buffer solutions pH 6.0 and 6.5 contained calcium chloride 0.05 M. The column was eluted by 1 column volume (CV) of eluents. The fractions at pH 4.2 and 4.8 were collected. The resin was regenerated by eluting with the 2 M sodium chloride solution.

The protein solution was stored at 4 °C. It was concentrated by flushing nitrogen through the solution. Amicon centrifugal filter units (Merck, Darmstadt, Germany) with nominal cut-off value of 10 kDa were used to change the solvent from the citrate buffer to water. The β-LG solution (pH 4.8 fraction) was lyophilized to dry mass. The powder was stored at −80 °C.

### 4.2. SDS-PAGE Analysis of Protein

Protein solution was analyzed by the SDS-PAGE method using Coomassie Blue R-350 staining and Invitrogen Bolt™ 4–12% Bis-Tris Plus polyacrylamide gel (Thermo Scientific, Waltham, MA, USA) with SeeBlue^®^ Plus2 Pre-Stained Standard (Thermo Scientific, Waltham, MA, USA) in non-reduced and reduced modes with, applying MES Running Buffer.

In case of water samples, protein solution with concentration of about 1 mg/mL was prepared. The protein solution was separated by the AF4 fractionation and was concentrated by flushing nitrogen through the solution. Amicon centrifugal filter units (Merck, Darmstadt, Germany) with nominal cut-off value of 10 kDa were used to change the solvent in the same way as described above. According to the manufacturer procedure (Thermo Scientific, Waltham, MA, USA)), proteins solutions were dissolved in a 2.5 µL Load Sample buffer (LDS), reduced and alkylated using the Sample Reducing Agent (10×) dithiothreitol (DTT) and iodoacetamide (IAA), respectively. Samples for analysis in a non-reduced mode were prepared by dissolving proteins fractions in the LDS buffer. The samples were then heated for 10 min at 70 °C and were introduced to the gel.

The electrophoretic process was performed at 200 V. After the separation process, the gel was stained for 20 min and destaining of the gel was carried out for 24 h in deionized water. The SDS-PAGE was performed also after the AF4-UV-MALS separation in order to confirm the purity of the fraction collected. The fractions were concentrated and analyzed as described above.

### 4.3. Characterisation of β-LG by MALDI-TOF/TOF MS Analysis

For this part of the study mass spectrometer MALDI-TOF/TOF (Bruker Daltonics, Bremen, Germany) equipped with a modified Nd:YAG laser operating at the wavelength of 355 nm and frequency of 2 kHz was used. The reagents used in the MALDI-TOF/TOF MS analysis were purchased from Sigma-Aldrich (Steinheim, Germany) with the highest commercially available degree of purity. The α-cyano-4-hydroxycinnamic acid (HCCA), 3,5-dimethoxy-4-hydroxycinnamic acid (sinapic acid—SA) and 2,5-dihydroxybenzoic acid (DHB) were used as a matrix and Protein Calibration Standard II for intact analysis and Peptide Calibration Standard II applied for the PMF study (all from Bruker Daltonics, Bremen, Germany) were used for calibration.

The spectrometric analysis for both intact and digested protein samples was performed in three stages. Stage I—gel electrophoresis according to the procedure described in Part 2.2 to check the purity of the obtained protein fraction. Stage II—the digestion of proteins in-gel with trypsin according to Bruker Proteomic protocols for mass spectrometry [80]. β-LG was dissolved in the buffer 10 mM ammonium bicarbonate (NH4HCO3, ABC) and incubated at 37 °C with trypsin overnight. Subsequently, the extraction of peptides was carried out by addition of 10 µL of 50% ACN with 1% TFA. Stage III—both intact and digested samples were applied to ground steel targets purchased from Bruker Daltonics (Bremen, Germany) using dry droplet method and spectrometric analysis.

The MS spectra of β-LG intact were recorded in a linear positive mode in the range of *m*/*z* 5000–100,000, while the peptide fingerprint mass spectra (PMF) of protein digested with trypsin was recorded in a reflectron positive mode in the range of *m*/*z* 500–3500. In both cases, the measurements were carried out at an accelerating voltage of 25 kV. To determine the fragment spectra, the laser-induced fragmentation technique (LIFT) in the same *m*/*z* range was used. The peptides obtained after the tryptic digestion of β-LG were identified using the BioTools software (Bruker Daltonics, Bremen, Germany). All data was collected manually, and the mass tolerance was set to 0.3 Da for the spectra and calibrated internally on immonium ions at laser power 80% and attenuation 27% for the MS/MS analysis.

### 4.4. Zeta Potential (ζ) Determination for β-LG

Different solutions at 0.09% (*w*/*v*) concentration were prepared: sodium and potassium chloride at pH range from 3.0 to 8.5 and sodium citrate, ammonium sulfate at pH range from 3.5 to 7.0 was also prepared. The sodium hydroxide, hydrochloric, sulfuric, or citric acids (1 M and 0.1 M acid and base solutions) (all reagents from Sigma-Aldrich, Steinheim, Germany) were added to the solution to obtain required pH values against pH meter. In this way, mixing of different ions was avoided. β-LG solutions at 0.4 mg/mL in all of these dispersants were prepared. The sample was loaded to DTS1070 cuvette (Malvern, Worcestershire, UK), and zeta potential was determined by Malvern Zetasizer NanoZS apparatus (Malvern, Worcestershire, UK).

Smoluchowski’s approximation (*f*(*κα*) = 1.5) in Henry’s equation (Equation (1)) was used [81]:

(1) $$\mu_{e}=\frac{2\varepsilon_{r}\epsilon_{O}\zeta f\left(\kappa \alpha \right)}{3\nu }$$

where *ε_r_* is the relative permittivity, *ϵ_O_* is the permittivity of a vacuum, *ζ* is the zeta potential (mV), *f*(*κα*) is Henry’s function, and *ν* is the viscosity (cP) of the liquid medium at the experimental temperature (25 °C). Measurements were performed in the automatic mode selection of the voltages and number of runs. Each measurement was performed in triplicate.

### 4.5. Characterization of β-LG by CE

The sample of β-LG was suspended in 0.09% citrate buffer at pH = 3.0, 5.0 and 7.0, respectively. CE analysis was performed using PA 800 plus (Beckman Coulter system, Brea, CA, USA) equipped with a DAD with the use of fused silica capillary (I.D. = 50 μm; Ltot = 67 cm; Leff = 50 cm; Beckman Coulter Inc., USA). Before use, a new capillary was rinsed with 0.1 M NaOH, deionized water, and the running background electrolyte (BGE) for 10 min. The protein samples were injected into the capillary with a pressure mode (3 psi, 5 s) and the analysis was performed at a constant voltage (10 kV) and the temperature of 23 °C. The sample at pH = 7 was analyzed in reverse polarity. The signal was monitored at λ = 214 nm. Between the runs, the capillaries were washed with 0.1 M NaOH, deionized water, and BGE for 2 min each. As the EOF marker, thiourea at 1 mg/mL concentration was used.

The electrophoretic mobility of β-LG during the CE assay was calculated from the Equation (2):

(2) $$\mu_{e}=\frac{L_{tot}L_{eff}}{V}\left(\frac{1}{t_{m}-t_{EOF}}\right)$$

where *μ_e_* is an electrophoretic mobility [cm2/V*s], *L_tot_* is the total length of the capillary [cm], *L_eff_* is the length to the detector [cm], *t_m_* is the migration time [s], *t_EOF_* is the EOF migration time [s], and V is the applied potential [V].

### 4.6. Characterization of β-LG by AF4-UV-MALS

Solutions of β-LG at the final concertation of 3 mg/mL were prepared in 0.09% (*w*/*v*) citrate buffer at pH 3.0, 5.0, and 7.0 values. The AF2000 Multi Flow system (Postnova Analytics GmbH, Landsberg am Lech, Germany) was used in this research. The used apparatus configuration has been the same as [45]. The specific refractive index increment value (dη/dc) of β-LG was set at 0.185 mL/g, and the extinction coefficient was of 0.960 mL/g cm. The UV detector acquires data at 280 nm. Berry model was used to calculate molar masses and the radius of gyration (Rg). Citrate buffer 0.09% at certain pH was used as a carrier liquid and prepared from the Milli-Q system (Merck Millipore, MA, USA)) and were filtered using 0.1 μm nylon membrane (Merck Millipore, Warsaw, Poland). All fractionation analyses were performed at room temperature. Conditions of AF4 fractionations were showed in Table 5, and the cross-flow program during elution is presented in Figure S8.

### 4.7. Characterization of β-LG by Far UV Circular Dichroism (UV–CD)

The sample of β-LG (0.3 mg/mL) was suspended in 0.09% citrate buffer at pH = 3.0, 5.0, and 7.0, respectively. Far UV circular dichroism (UV–CD) was performed using J-815 spectrometer (JASCO, Cremella (LC), Italy). The experimental conditions were as follows: sensitivity: high, response: 1 sec, band width: 1 nm, scanning speed: 50 nm/min, accumulation: 10, data pitch: 0.2 nm. All data were background-corrected against citrate buffer in certain pH. All data were acquired in the range from 300 to 190 nm at room temperature using the quartz cell with a path length of 0.02 cm. For estimation of the secondary structural composition, the ECD spectra were submitted to the JASCO Secondary Structure Estimation (SSE) software based on the Principal Component Regression method (PCR). The multivariate analysis allowed us to obtain quantitative data of α-helix, β-sheet, turns, and random coil contents from experimental CD spectra.

## 5. Conclusions

The β-LG was isolated from the WPI solution. The fast flow column chromatographic technique was used. The electrophoretic purity of the isolated protein was achieved. The MALDI-TOF/TOF MS analyses assisted for the identification of protein. Additionally, the matrices used in the studies allowed us to distinguish the protein isoform A and B. The pH correlation with the zeta potential in four solvent compositions was studied. The medium components have significant impact on the protein stability and the isoelectric point. The capillary electrophoresis with the UV detection was applied for the protein oligomerization screening. Obtained data were further confirmed by AF4-UV-MALS. Multi-angle light scattering was used to characterize the self-association of β-LG in citrate buffer at pH: 3.0, 5.0, and 7.0. The monomer was present at acidic condition. At pH 5.0, the forms from dimer to octamer were observed, wherein the AF4 system did not separate these forms into individual fractions. The equilibria also depend on the protein concentration. At pH 7.0, the β-LG forms a dimer. The SDS-PAGE and MALDI-TOF/TOF MS analyses after the AF4 separation process were used to identify the purity of the fraction. It showed that the separation process did not have a negative impact on the protein structure. The far circular dichroism (CD) spectra carried out at pH 3.0, 5.0, and 7.0 confirmed that the β-sheet conformation is dominant at pH 3.0, 5.0, while at pH 7.0 this conformation is approximately in the same quantity as a-helix and random structures.

## Figures and Tables

**Table 1:** Masses obtained for HCCA and SA matrices using MALDI TOF/TOF MS in the intact approach.

		pH 4.5		pH 5.0		pH 5.5		pH 6.0	pH 6.5
		Mass [kDa]							
HCCA	β-LG	18.348 ± 0.137		–		–		–	–
	α-LA	14.173 ± 0.137		14.184 ± 0.137		–		–	–
SA	β-LG	18.301 18.382	±0.000	–		–		–	–
	α-LA	14.197 14.415	±0.000	14.190 14.399	±0.000	14.199 14.417	±0.000	–	–

**Table 2:** MS and MS/MS identification of β-lactoglobulin peptides isolated from WPI.

Mass [Da]		Intensity	Sequence Range	Sequence from MS/MS
Measured	Theoretical			
837.527	837.476	650.565	158–164	ALPMHIR
1121.531	1121.468	335.855	77–85	WENGECAQK
1193.758	1193.678	188.018	108–117	VLVLDTDYKK
1245.658	1245.584	262.086	141–151	TPEVDDEALEK
1635.868	1635.775	1016.811	141–154	TPEVDDEALEKFDK
1715.902	1715.806	5757.401	165–178	LSFNPTQLEEQCHI
2313.428	2313.259	2143.636	57–76	VYVEELKPTPEGDLEILLQK
2355.446	2355.365	220.694	87–107	IIAEKTKIPAVFKIDALNENK
2707.574	2707.376	2194.688	31–56	VAGTWYSLAMAASDISLLDAQSAPLR
2846.512	2846.480	431.587	155–178	ALKALPMHIRLSFNPTQLEEQCHI

**Table 3:** Migration times (tm, min), electrophoretic mobility (µe, cm^2^/Vs) of β-lactoglobulin obtained at different pH values.

Migration Time (*t_m_*, Min)			Electrophoretic Mobility (µ_e_, cm^2^/V_s_)		
pH = 3	pH = 5	pH = 7	pH = 3	pH = 5	pH = 7
15.16	12.89	1.95	0.003	0.100	−0.001
	15.20	11.99		0.059	0.004
	16.42	12.39		0.049	0.003
	16.75	17.42		0.047	0.001
	16.90			0.046	
	17.29			0.043	
	17.60			0.042	
	19.25			0.035	
	19.83			0.033	

**Table 4:** Mean of weight average molar mass, radius of gyrations, and protein content in the first fraction at pH 3.0, 5.0, and 7.0 from AF4–UV-MALS analyses.

Analysis at pH Buffer	Radius of Gyration [nm]	Mw Average [kDa]	Mass of Fraction from UV [µg]
3	45.1 ± 4.1	22.1 ± 0.5	230 ± 41
5	83.0 ± 35.8	149.0 ± 13.7	176 ± 63
5	115.1 ± 18.4	50.2 ± 12.5	325.2 ± 28.1
7	37.2 ± 7.9	38.7 ± 8.2	161 ± 23

**Table 5:** The focusing parameters of method at three experimental conditions.

pH	Focus			
	Injection Flow [mL/min]	Injection Time [min]	Initial Cross Flow [mL/min]	Transition Time [min]
3.0	0.1	5.0	3.0	1.0
5.0	0.2		1.0	3.0
7.0	0.1		1.0	2.0

## Supplementary Materials

The following are available online at https://www.mdpi.com/1422-0067/21/24/9711/s1.
