# Supplementary material for: Isolation and Self-Association Studies of Beta-Lactoglobulin
Source: Int J Mol Sci. 2020 Dec 19;21(24):9711. doi: 10.3390/ijms21249711 (PMC7766286; doi:10.3390/ijms21249711)
Supplement: Supplementary file 1 [file ijms-21-09711-s001.pdf]

Isolation and Self-Association Studies of Beta-Lactoglobulin

A. Gołębiowski[1, 2], P. Pomastowski[1], A. Rodzik[1, 2], A. Król-Górniak[1, 2], T. Kowalkowski[1, 2] M. Górecki[3], and B. Buszewski[1, 2*]

[1] Centre for Modern Interdisciplinary Technologies, Nicolaus Copernicus University in Torun, 4 Wileńska St., 87- 100 Torun Poland

[2] Department of Environmental Chemistry & Bioanalytics, Faculty of Chemistry, Nicolaus Copernicus University in Torun, 7 Gagarina St., 87- 100 Torun Poland

[3] Institute of Organic Chemistry, Polish Academy of Sciences, 44/52 Kasprzaka St., 01-224 Warsaw, Poland

* Corresponding author: bbusz@umk.pl; Tel: +48 (56) 665-60-38

Supplemented tables and figures to the manuscript

Table S1. β-LG masses for three fractions with AF4-UV-MALS using MALDI-TOF/TOF MS.

			**Fraction 1**		**Fraction 2**		**Fraction 3**	
					**Mass of β-LG [kDa]**			
**HCCA**	pH 3	Monomers	18.356		18.362		18.362	
		Dimers	36.639		36.661	± 0.066	36.661	± 0.066
		Trimers	55.029	± 0.066	55.024		55.018	
		Tetramers	73.387		73.389		73.374	
		Pentamers	91.732		–		–	
	pH 5	Monomers	18.371		–		–	
		Dimers	36.737		–		–	
		Trimers	55.109	± 0.308	–		–	
		Tetramers	73.493		–		–	
		Pentamers	91.834		–		–	
	pH 7	Monomers	18.339		–		18 341	± 0.000
		Dimers	36.675		–		36 718	
		Trimers	55.051	± 0.000	–		–	
		Tetramers	73.421		–		–	
		Pentamers	91.636		–		–	
**DHB**	pH 3	Monomers	18.280<br>18.369	± 0.088	18.364<br>18.280	± 0.088	18.369<br>18.281	± 0.088
		Dimers	–		–		–	
		Trimers	–		–		–	
		Tetramers	–		–		–	
		Pentamers	–		–		–	
	pH 5	Monomers	18.291<br>18.380		–		–	
		Dimers	36.741	± 0.000	–		–	
		Trimers	55.025		–		–	
		Tetramers	73.460		–		–	
		Pentamers	91.838		–		–	
	pH 7	Monomers	18.289<br>18.377		–		18.294<br>18.379	
		Dimers	36.742		–		36.736	± 0.000
		Trimers	55.025	± 0.000	–		55.090	
		Tetramers	73.384		–		73.416	
		Pentamers	91.793		–		–	

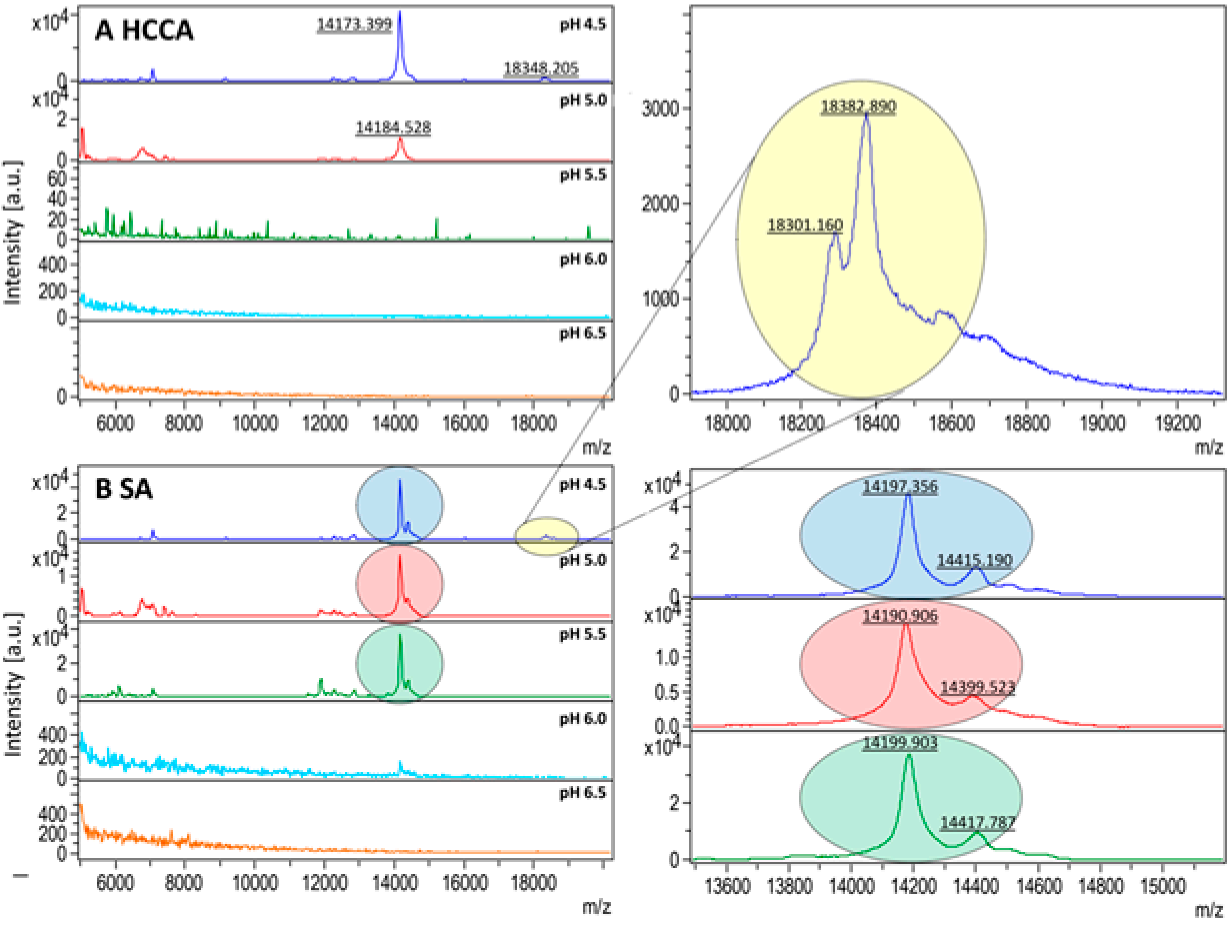

Fig. S1 MALDI-TOF/TOF MS spectrums of intact proteins. Two matrices: α-cyano-4-hydroxycinamic acid (HCCA) – A and 3,5-dimethoxy-4-hydroxycinnamic acid (sinapic acid, SA) – B were used.

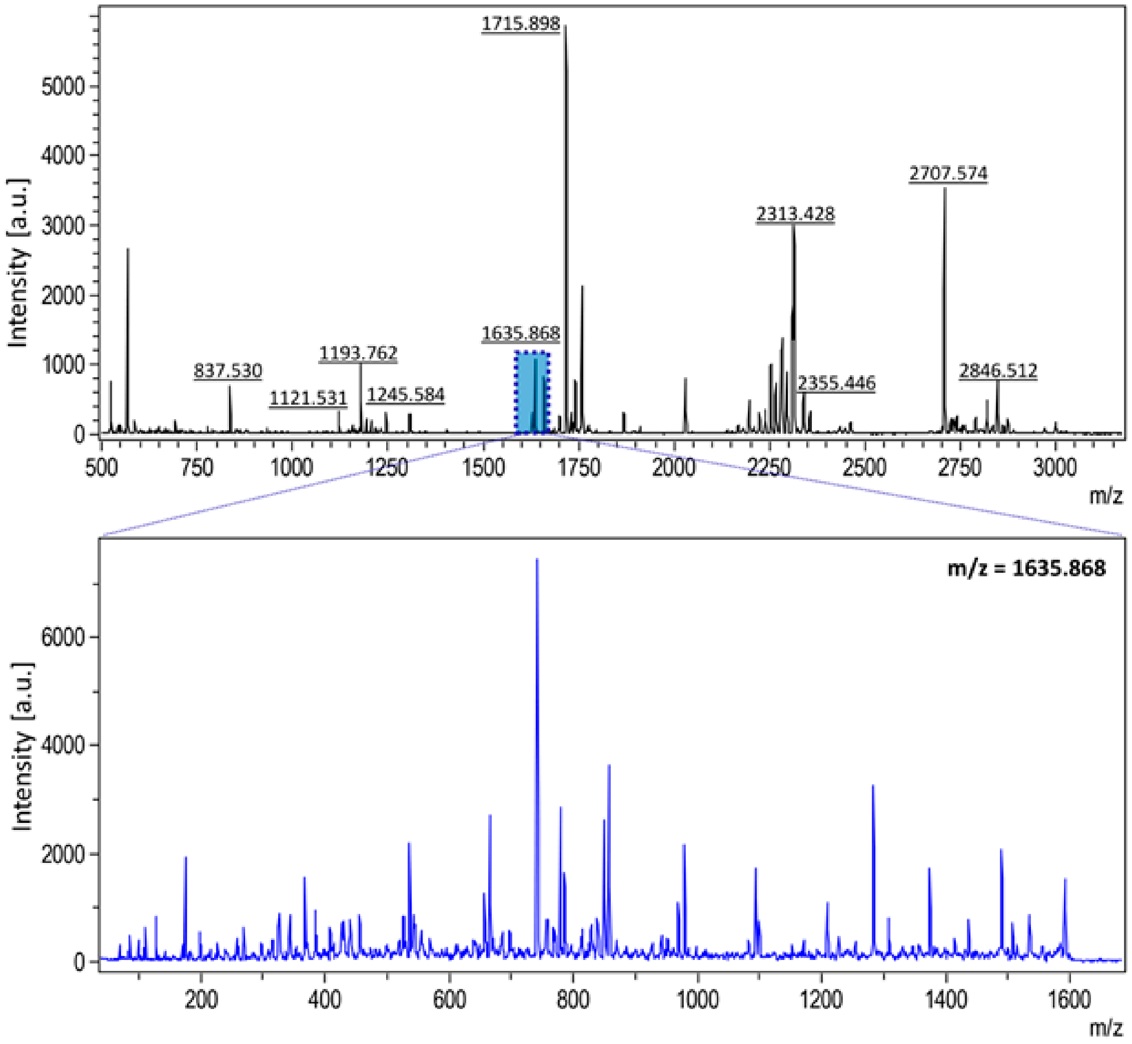

Fig. S2 MALDI-TOF/TOF MS spectrum of tryptic digests of protein sample. Sample peak at m/z 1635.868 was investigated.

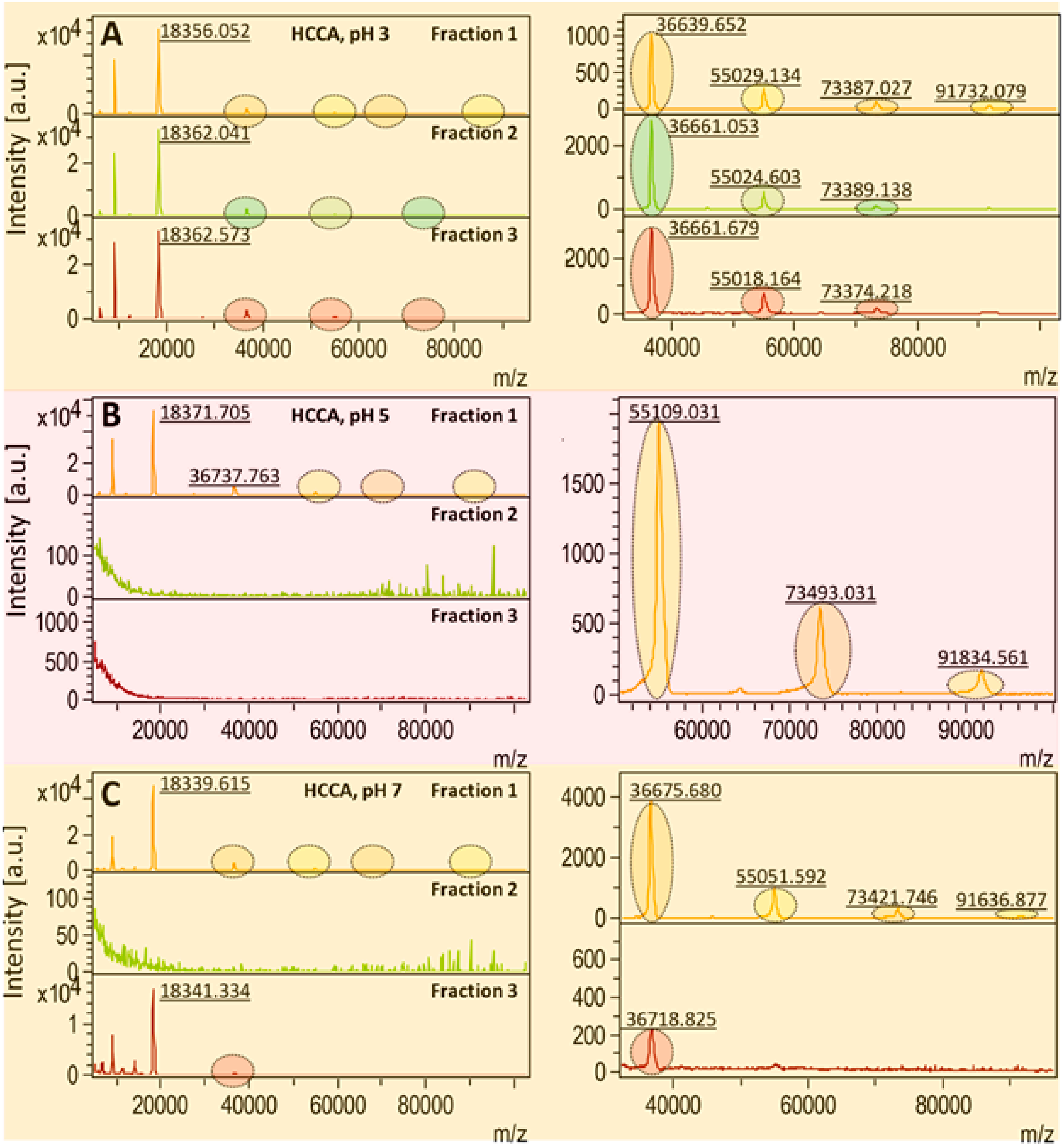

Fig. S3 MALDI-TOF/TOF MS spectrums of intact proteins. Two matrices: α-cyano-4-hydroxycinamic acid (HCCA) – A and 3,5-dimethoxy-4-hydroxycinnamic acid (sinapic acid, SA) – B were used.

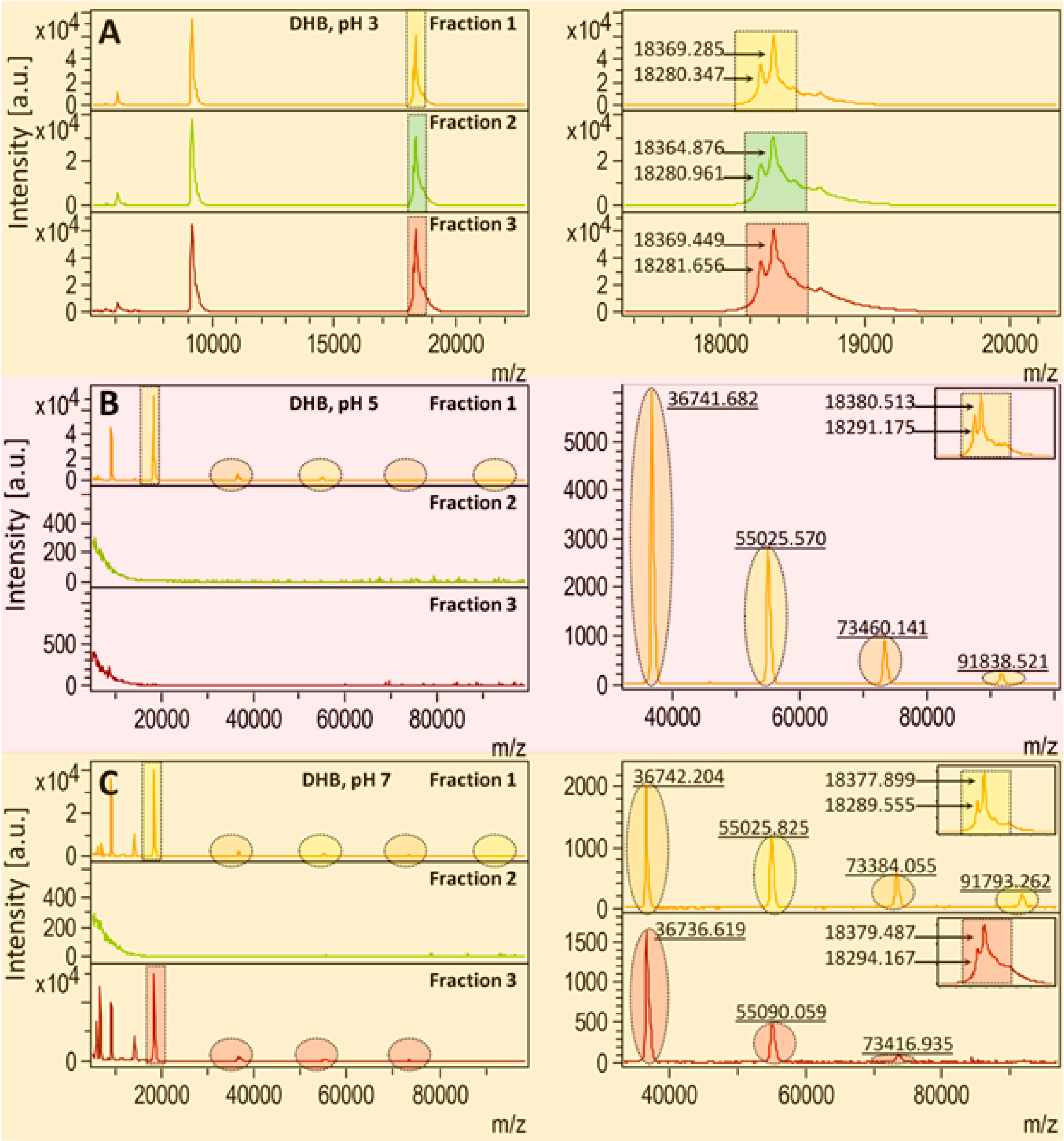

Fig. S4 MALDI-TOF/TOF MS intact protein analyses of three fractions after AF4-UV-MALS characterization. The DHB matrix was shown.

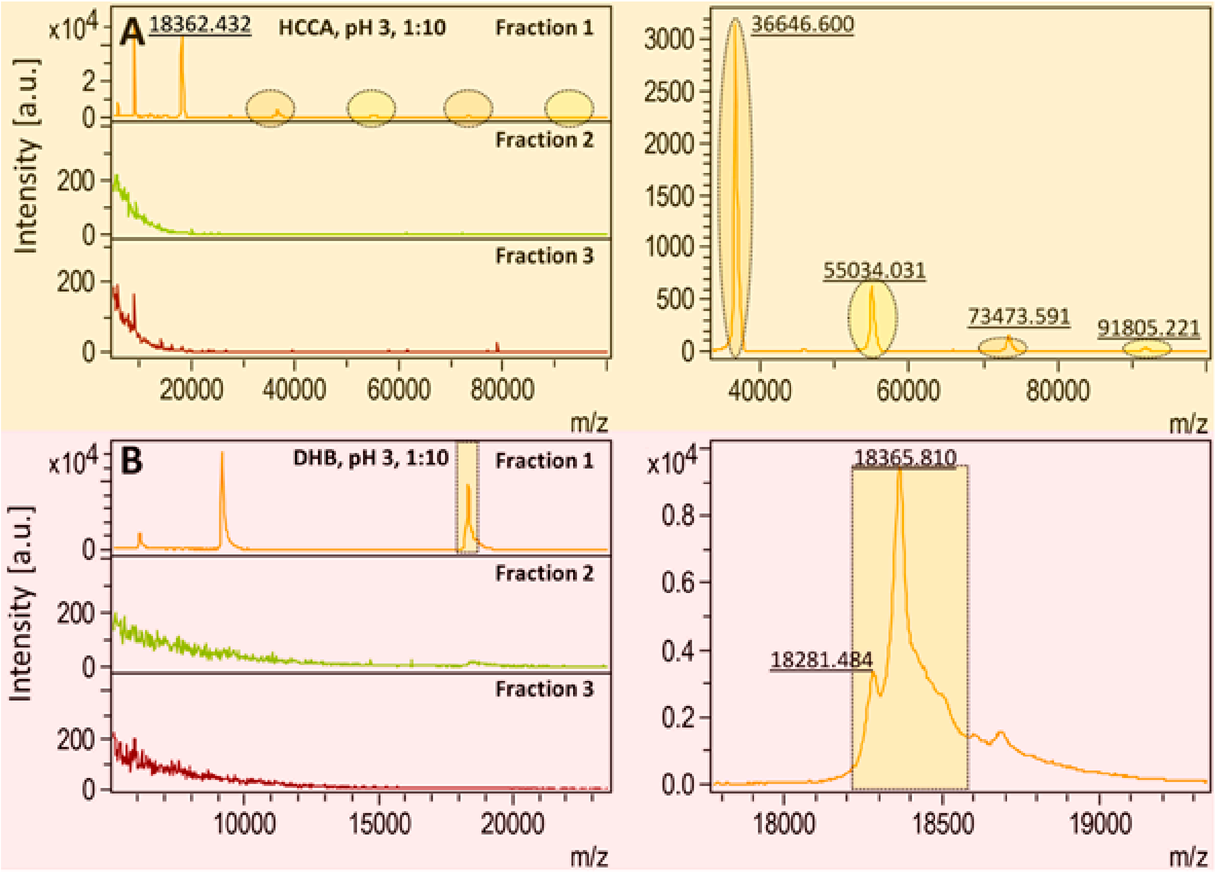

Fig. S5 MALDI-TOF/TOF MS intact protein analyses of three fractions after AF4-UV-MALS characterization. Comparison of the matrixes.

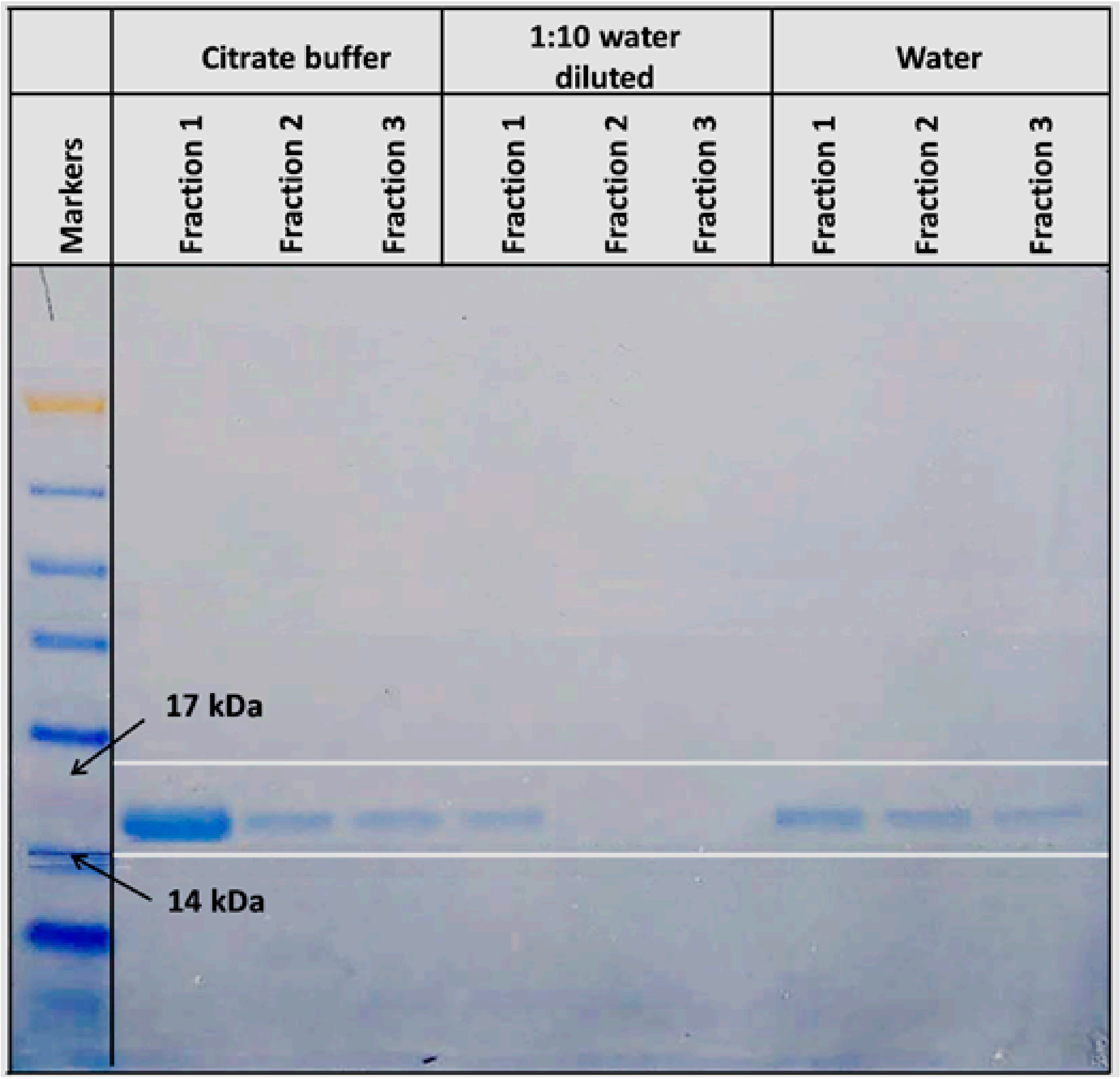

Fig. S6 The SDS-PAGE electropherogram of β-LG fractions after the AF4-UV-MALS characterization in 0.09% citrate buffer at pH 3.0. From the left to the right, fractions in citrate buffer, diluted with water 1:10, and water samples (after desalting step on Amicon membrane).

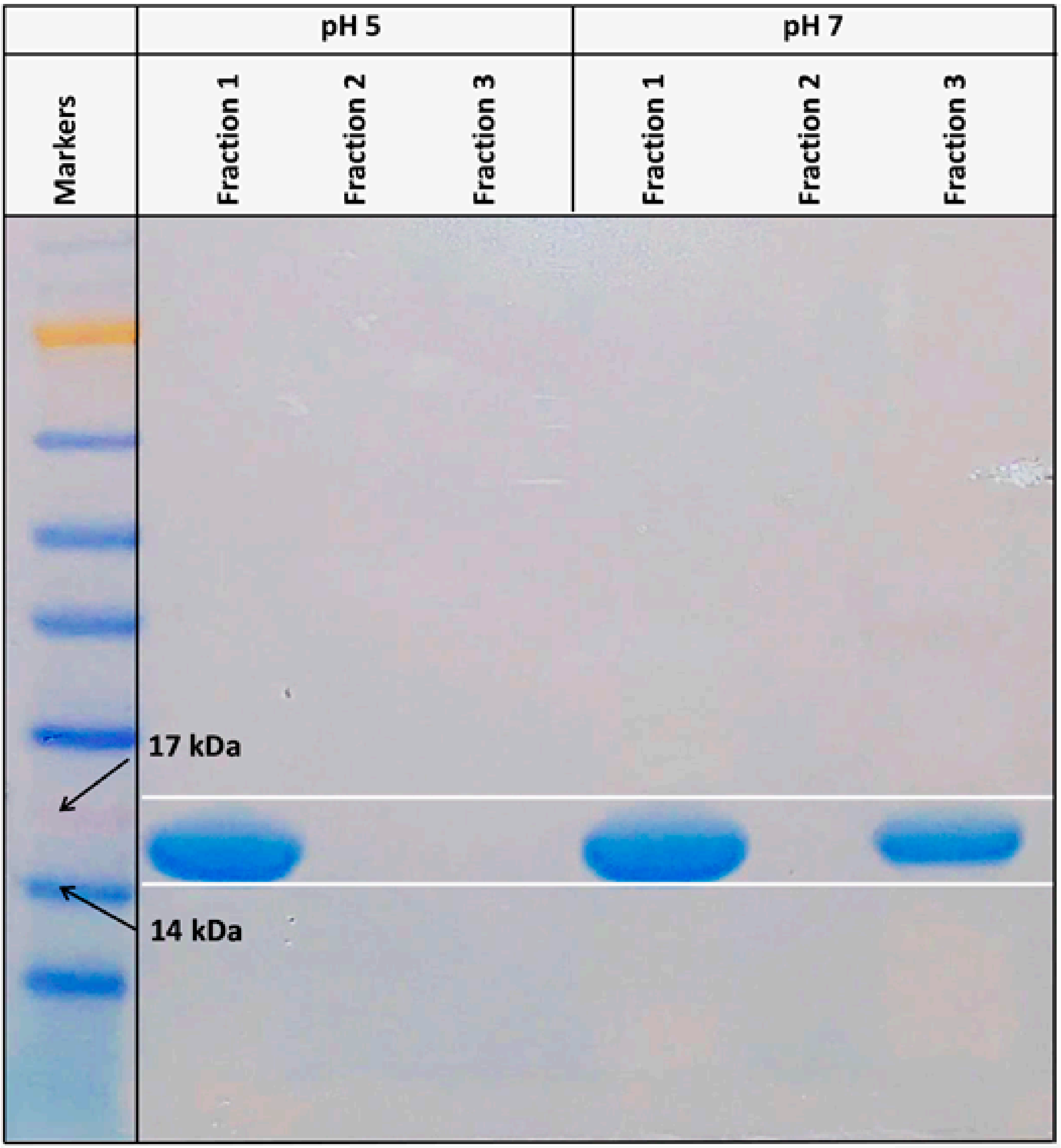

Fig. S7 SDS-PAGE of three fractions of the protein samples at pH 5.0 and 7.0 from AF4-UV-MALS characterization.

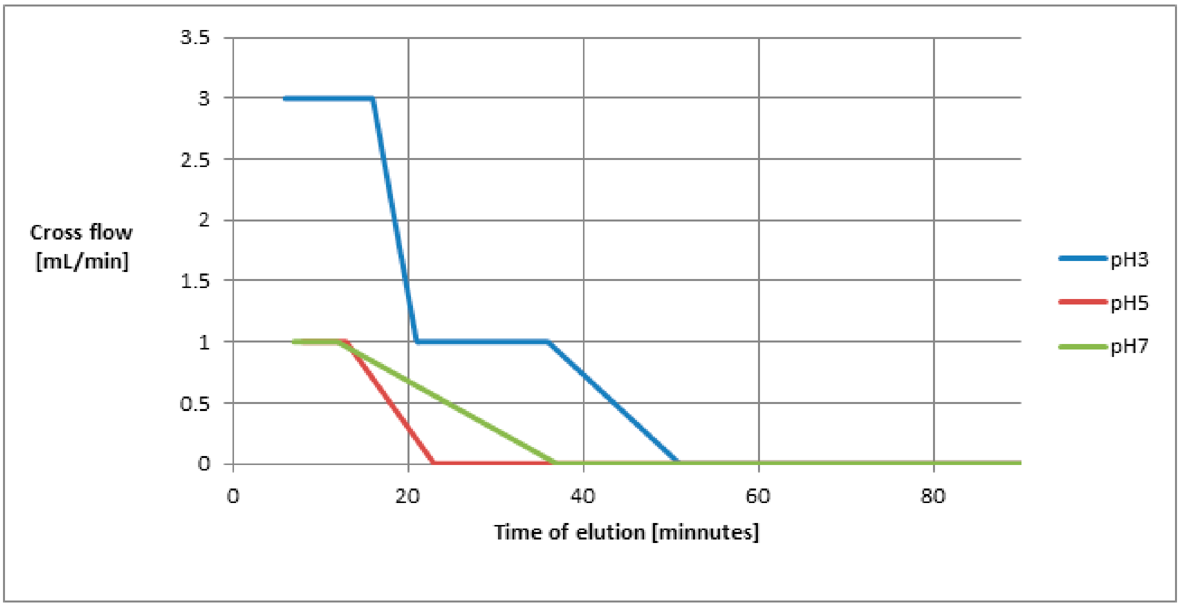

Fig. S8. The cross flow gradients during the elution at different pH.
